# Video Sensor Architecture for Surveillance Applications

**DOI:** 10.3390/s120201509

**Published:** 2012-02-03

**Authors:** Jordi Sánchez, Ginés Benet, José E. Simó

**Affiliations:** Instituto de Automática e Informática Industrial (*ai2*), Universidad Politecnica de Valencia, C/. de Vera, s/n, E-46022 Valencia, Spain; E-Mails: jorsanpe@gmail.com (J.S.); jsimo@disca.upv.es (J.E.S.)

**Keywords:** image processing, computer vision, real-time systems, domain-specific architectures, distributed smart cameras, surveillance systems

## Abstract

This paper introduces a flexible hardware and software architecture for a smart video sensor. This sensor has been applied in a video surveillance application where some of these video sensors are deployed, constituting the sensory nodes of a distributed surveillance system. In this system, a video sensor node processes images locally in order to extract objects of interest, and classify them. The sensor node reports the processing results to other nodes in the cloud (a user or higher level software) in the form of an XML description. The hardware architecture of each sensor node has been developed using two DSP processors and an FPGA that controls, in a flexible way, the interconnection among processors and the image data flow. The developed node software is based on pluggable components and runs on a provided execution run-time. Some basic and application-specific software components have been developed, in particular: acquisition, segmentation, labeling, tracking, classification and feature extraction. Preliminary results demonstrate that the system can achieve up to 7.5 frames per second in the worst case, and the true positive rates in the classification of objects are better than 80%.

## 1. Introduction

Image-based sensing is currently a powerful source of data in many application areas such as surveillance, monitoring and automation. Images should be processed in order to extract application-driven data but centralized processing is not feasible in the presence of large amounts of cameras distributed along big geographical areas. In this scenario, when processing limitations and bandwidth availability are important restrictions, the current tendency is to use *embedded video processing* nodes. Each node processes its own image stream, extracting the required application data, taking into account not only the functionality required but also the resource availability (processing power and available bandwidth) and QoS acceptance levels. Obviously, in a network of connected video processing nodes, each node has different implementation requirements. Sometimes the video stream should be transmitted in order to allow a human operator to watch it. In other cases the video stream is not required or it is simply not possible to send it (*i.e.*, when only low bandwidth wireless communication is available). In general, the application requires different processing algorithms to be applied to the captured images. The general assumption is to have a network of nodes, each one extracting different data from captured images. This assumption has an important impact on the node architecture definition. The node architecture should be flexible in terms of hardware and software configuration, allowing to handle the inherent heterogeneity while providing a common application programming and deploying framework.

Distributed smart cameras have received increased focus in the research community over the past several years [1–3]. In [4] can be found an interesting survey of the main technological aspects of advanced visual-based surveillance systems. In [5], a detailed description of a smart camera, called SmartCam and a distributed video surveillance system can be found. Foresti in [6] describes the low-level video processing requirements for the implementation of smart cameras. In [7–9], the tracking people problem is discussed and some applications are described. The existence of visual sensing nodes with increasing DSP processing capabilities, together with the possibility of being interconnected through wireless communication links opens up a new realm of intelligent vision-enabled applications. In [10], a case studio where DSP technology can be used to enhance the real-time response is described.

Real-time image processing and distributed reasoning capabilities not only enhance existing applications but also instigate new applications [11–14]. Potential application areas range from home monitoring and smart environments to security and surveillance in public or corporate buildings or ubiquitous sensors networks for urban environments [3,15]. Critical issues influencing the success of smart camera deployments for such applications include reliable and robust operation with as little maintenance as possible.

The architecture described in this paper is part of the SENSE research project [16], which undertakes the task of developing a distributed intelligent network of sensory units that process images in order to describe their environment in a cooperative way, aiming at surveillance purposes. This project has been conceived as a multi-layer architecture. Data flows from lower layers to upper layers, processing higher level data on each step, until real knowledge about the state of the environment is obtained. In [17] and [18] some preliminary results obtained in this project were presented. In this paper, we present the details of the architecture involving the lowest level layers, which provides object detection, classification and tracking analysis to upper layers. Due to this multi-level architecture, the low level processing has some requirements, namely, correct detection of objects, good classification rates and a minimum frame rate to achieve consistent tracking. All the documentation related with this project, as well as the hardware schematics and sources of all the developed video processing software, can be found in the web repository of the project (http://www.sense-ist.org).

This paper is organized as follows. In Sections 2 and 3, the embedded architecture is presented and some of its main features are discussed. In Section 4, we present the embedded vision system implemented. Finally, in Section 5, the obtained results from real tests are discussed.

## 2. Hardware Architecture Description

The Embedded Video Subsystem (EVS) is a specialized sensory component of the overall architecture of the SENSE node. It acquires and processes video images, extracting features in a modal way. Extracted features are sent to the Reasoning and Decision-making Unit (RDU), which is in charge of detecting the state of the node surroundings. This video subsystem offers an interface with higher level software to control the modality, QoS and other special features.

Our video board has been designed using two Blackfin DSP processors for video processing tasks and a Spartan III FPGA, which provides a way of configuring hardware outline. This cooperation between DSP and FPGAs is very interesting and has been also reported recently in different applications related to video processing [13,14,19–22].

**Figure 1.**
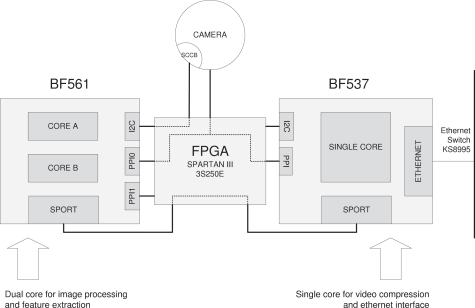
Video Board Hardware Outline.

Figure 1 depicts the outline of the video board of the SENSE node. The architecture has been designed so that processor BF561 is dedicated to processing the images and performs the feature extraction. The obtained features are then sent to BF537, which works as a bridge, propagating the features to the RDU or any other suitable client. Processor BF537 also is in charge of compressing the images and sending them. Both image and feature streams are sent to the RDU by the BF537 via its Ethernet device.

Features and compressed images are obtained on different processors. As each processor has its own clock, a mechanism for aligning features and compressed images has been implemented. In order to align them, the FPGA writes a time stamp (Despite the name of the term *time stamp*, it is not intended for timing purposes, but only for aligning XML features with their corresponding compressed image.) on the incoming images from the camera, simply replacing the first bytes of the frame with the time code. This does not affect neither the processing nor the compression of the images.

Blackfin core modules provides a PPI port to connect digital cameras following YUV, YCbCr and RGB standards. The camera programming can be done through I^2^C interface using the Serial Camera Control Bus (SCCB) standard. The current choice for the camera is to use an Omnivision OV7660 VGA camera because image processing for higher resolutions is too heavy for these Blackfin DSP processors given the application QoS requirements. One constraint for this system is that it works with a YUV 422 interface, which affects the segmentation process. Both image analysis and compression algorithms use this color space.

In our scheme, the FPGA interface is responsible for pin assignments through all the board. This approach enhances the versatility, allowing multiple changes on the hardware without modifying the board or even the software.

## 3. Software Architecture Description

### 3.1. Feature Extraction Software Architecture

To achieve the minimum required frame rate, attention must be paid to optimize the system performance without losing Quality of Service. In this case, parallelization as well as a good memory management have been crucial to obtain a good performance.

**Figure 2.**
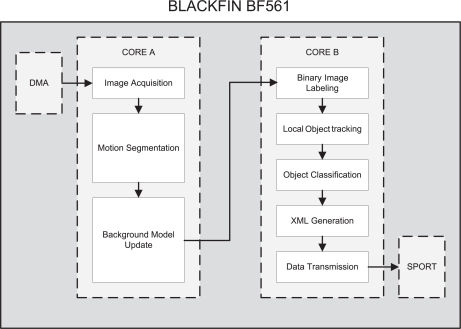
Video processing algorithm overview.

As seen in Figure 2, the process can be understood as a software pipeline. Boxes with dashed lines depict processing units that can work in parallel. Each phase feeds the next one with the data it has processed. The preliminary designs for this system included FIFO buffers between phases, where the intermediate data are stored. This approach has the advantage of being very easy to implement. However, we found two main disadvantages on this approach:

1. High latency. When a phase runs faster than the next one, the interconnection buffer gets full. This means that the data will have to wait for longer queues, increasing system latency. This does not affect the frame rate but increases the time delay between the capture and the feature transmission.
2. Data destruction. In our system, it is important for the last phases to get access to some data stored in the first phases of the system (*i.e.*, the original frame). However, due to the large number of intermediate processes, for the time the last phases access the data stored previously during the first ones, this data may have been replaced.

**Figure 3.**
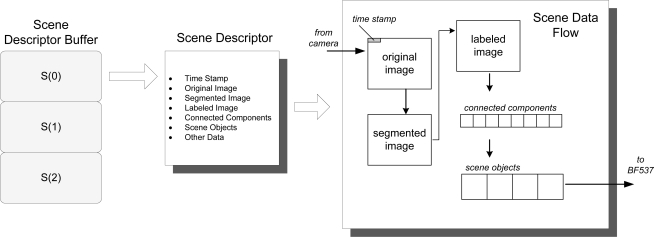
Scene Descriptor outline and information flow.

To solve these two problems, a segmented processor based pipeline has been implemented. First, a structure has been created to hold all the information relative to a single frame, including original image, segmented image, labeled image, objects on scene and some extra metadata (for example, time stamp and number of objects) which we call *Scene Descriptor* (SD). Figure 3 depicts the structure of this block and how the information flows and is transformed. Note that the individual elements of the *Scene Descriptor Buffer* are denoted as *S*(*k*), being *k* the number of the processing cycle. The original image is processed in order to obtain a segmented image which contains the foreground pixels found. Then, the image is labeled in order to detect and classify the connected components, *i.e.*, regions of pixels which are 8-connected. Connected components are filtered and processed to detect scene objects, which are the elements containing the high level data about the objects found (tracking information and classification probabilities among others).

We have three parallel execution units on the BF561 processor that are useful for our purposes: (1) the DMA device; (2) core A; and (3) core B. All these units can work concurrently so the system can process, at most, three different SDs at the same time. So, the point of view is that we have several SDs being processed, and each SD is at a different phase of the process.

**Figure 4.**
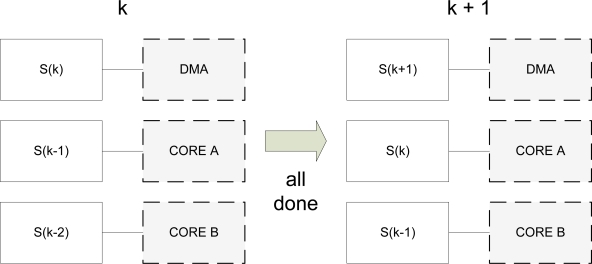
Scene Description Table assignment to processing units on the vision pipeline.

The synchronization mechanism for this algorithm is a very important issue. Each processing unit operates with a single frame until it finishes. Then, it has to wait, if necessary, for the other units to finish. When all the processing units have finished their corresponding tasks, each SD advances to the next unit and the system goes to a new processing cycle. Figure 4 depicts the process. On cycle *k*, *S*(*k*) is entering the system. This means that the DMA device is receiving an image from the camera and is storing it in memory. When all the units finish their corresponding tasks, the system goes to a new cycle. Then, *S*(*k*) is assigned to core A of the BF561 to execute the appropriate operations.

With this approach we address the above mentioned two problems, namely, high latencies and data destruction. First, the latency can be measured and reduced to a minimum, as there are no queues in the system. Also, the data destruction problem is solved because all the data related with a given image is stored in a centralized structure and remains on the system during the whole process.

### 3.2. System Services and Interface

A Blackfin BF537 processor is being used for interfacing purposes. This processor enables an Ethernet interface to offer three main services: image compression and streaming, a bridge to send high level XML image description data produced by processor BF561 and a node configuration service.

It is intended that the compressed video is sent to a centralized unit, which may be supervised by an operator. In order to reduce the network overload due to the high amount of data to send, each video frame is compressed to JPEG format. So, frames will be sent in a way that may require further processing to create video movies. Other video encoders can be attached in the system but this enhancement is out of the scope of the developed prototype.

**Figure 5.**
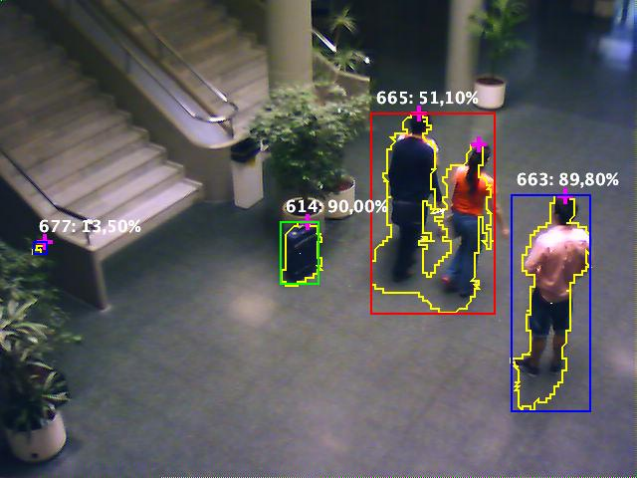
Image obtained from a client application showing the image as well as other information provided by the low level video processing services. The objects are identified with a label, followed by the membership probability. The membership is coded with the color of the bounding boxes (green: Luggage, red: Group, blue: Person). Contours of the objects are also painted, as well as heads, which are depicted by red crosses.

A JAVA client application has been developed to test the prototype. This client is prepared to connect to the platform via BF537 processor. It makes use of the three services above mentioned. The configuration service has proved to be very useful in the tuning of the parameters of the platform. Figure 5 shows an image obtained with the JAVA client during a real test, and the result of merging the separate JPEG compressed images and XML messages.

## 4. Image Processing and Feature Extraction

As many surveillance systems, the video processing algorithm is based on background learning and subtraction. A model of the background (an image scene without objects) is obtained and stored. Incoming images are compared with this background model in order to detect environment changes. These changes are then binarized and analyzed to extract objects of interest. Finally, the obtained scene description is coded into an XML message and the results are sent to the BF537 via serial port. Figure 2 depicts the overview of the algorithm as well as the distribution of the software across the processing units.

### 4.1. Image Acquisition

To acquire an image from the camera we just need a function to trigger the *DMA receive* operation. This is done through an execution thread hosted on core A, which performs two tasks: it triggers the *DMA transmission*, and recovers the time stamp from the incoming image.

In order to simplify the system design, the FPGA attaches the time stamp of each frame as a watermark on the first bytes of each image. This way, both processors receive exactly the same information from each frame, so there is no need to perform additional synchronizations to correlate compressed images and high-level data extracted from them. Recovering the time stamp is simply reading the first bytes of the image, where it was recorded by the FPGA. The time stamp modifies the image in a way that is imperceptible and does not affect the vision process.

### 4.2. Motion Segmentation and Background Update

Objects in a captured frame are detected by comparing the incoming images with an empty scene model known as *background model* or *environment model*. This model represents the scene completely empty of foreground objects (*i.e.*, an empty corridor). By comparing the incoming images with the background model, it is possible to detect regions of the image where there have been changes. This process is known as image segmentation. For each pixel on the image, we measure the rate of variation with the background model. If the variation exceeds a threshold, the pixel is considered as foreground. Thus, the resulting segmented image is a binary image.

The process of creating the background model consists of acquiring several images and perform a median operation for each pixel array to get the most probable value for that pixel. This process produces very good results, and the background model can be inferred even on a scenario where objects are moving in front of the camera. The more images we acquire for modeling the background, the more quality it is going to have. However, there are limited memory resources on the processor so we have an upper bound for the number of images we can store. Due to this, the amount of video that the processor can record is quite low (about three seconds). A stationary foreground object can interfere with the environment model and the vision algorithm would then be affected. The solution we have adopted is to expand the acquisition of the images over the time by including a stride parameter on the background creation algorithm. Thus, being *s* the stride, we only take one of each *s* frames, and then perform the median of all pixel arrays for the stored images. This reduces even more the impact of transiting objects during the background creation process.

In order to detect foreground objects, color segmentation is being used. We have taken profit of the YUV color space to implement a very efficient color segmentation. YUV color space has the advantage of having separate luminance and color coordinates. Thus, we can take advantage of this fact to analyze pixels both by its luminance variation and by its color variation.

We check the luminance variation (Y coordinate) to decide whether the pixel is foreground or background. In our tests, we stated that a luminance decrease can be produced either by an object or by a shadow. On the other side, a luminance increase is more likely to be produced by a transiting object than anything else. With these assumptions in mind, we use two different thresholds, one for a luminance increment which we have called upper threshold, and another for a luminance decrement, which we have called lower threshold. So, in order to avoid detecting shadows as objects, the lower threshold is more restrictive than the upper threshold.

If the luminance variation of a pixel exceeds neither the upper nor the lower threshold, we check the color variation with the U and V values. Color coordinates on the UV space cannot be separated. Instead, they belong to a two dimensional bounded space where each point stands for a different color. Therefore, color variation is obtained as the two dimensional distance between the UV coordinates of the image and the UV coordinates of the background model. Equations (1), (2) and (3) model the motion segmentation for the upper, lower and color thresholds:

(1) $$\frac{I_{t}(Y,i,j)-B_{t}(Y,i,j)}{B_{t}(Y,i,j)}>T_{u}$$

(2) $$\frac{I_{t}(Y,i,j)-B_{t}(Y,i,j)}{B_{t}(Y,i,j)}<T_{l}$$

(3) $$|I_{t}(U,i,j)-B_{t}(U,i,j)|+|I_{t}(V,i,j)-B_{t}(V,i,j)|\;\;>T_{c}$$

where *T_u_*, *T_l_* and *T_c_* are the upper, lower and color thresholds (heuristic values obtained from experimental data taken in previous recordings), *B_t_*(*X, i, j*) is the value of coordinate X of background model pixel (*i, j*) at time *t* and *I_t_*(*X, i, j*) is the value of coordinate X of current frame pixel (*i, j*) at time *t*. If any of the equations above are true, then the pixel (*i, j*) is considered to be a foreground pixel. If the value of the pixel does not exceed any threshold, then it is considered to be a background pixel. Notice that, for Equation (3), we are using the Manhattan distance and not the Euclidean distance. The Euclidean distance is very heavy for the processor, as it involves calculating a square root for each pixel on the image. Despite not being an exact distance, Manhattan distance has proved to work quite well and avoids complex operations. Also, this enhances notably the timing performance of the system.

The system has to take into account slow illumination variations like those produced by the sunrise or the sunset. Illumination variations affect the detection of foreground objects to a point that the entire image could be detected as foreground. To absorb these variations, the environment model must be updated regularly.

To update the background model, we have developed a new background update algorithm which is based on continuous background model learning. The system is continuously obtaining the most probable value for each pixel from a series of samples using a median filter. As this process is very heavy for the processor, the update is performed across several frames.

**Figure 6.**
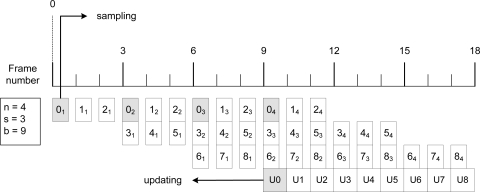
Background model update process by continuous background model learning.

The structure of this algorithm is as follows. The images are divided into several blocks. For each incoming image, we take a sample of one or several blocks of pixels. When we have enough samples of a block, we update it by obtaining the median of each pixel it is composed. Only one block can be updated at the same system cycle. Figure 6 depicts this process with an example. Three parameters configure the algorithm: *n*, which is the number of samples we take for each pixel, *s*, which is the stride (as described above), and *b*, the number of blocks in which we divide the image. In Figure 6, each sampled block is marked as *X_Y_* to refer to the sample *Y* of block *X*, and the updating blocks as *UX* to refer to the update of the block *X*. With this process, all the background parameters are met. As can be seen in this example, for each block, 4 samples are taken, and the distance between samples is 3 frames.

**Figure 7.**
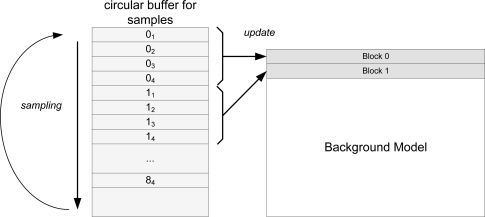
Background model update spatial outline.

Samples are collected into a circular buffer. From this buffer, all the data has to be indexed with correctness. Figure 7 depicts background update algorithm spatial scheme. Due to memory constraints, not all the samples can be stored at the same time, which is why we use a circular buffer. Once a block has been updated, the memory its samples occupy on the circular buffer is no longer needed. This memory can be reused to store more blocks.

The total time needed by a block to be updated is *n × s* frames, which is the time the samples of the block will remain in the circular buffer (we will refer to this as *active* block). Given that the time a block will remain active is *n × s*, at time *n × s* + 1 this block can be removed and its memory recycled. With this value, we can obtain the minimum size of the circular buffer. As can be seen in Figure 6, for each new image, a new block gets active and enters the circular buffer. So, the maximum number of active blocks will be *n × s*. The minimum size of the circular buffer is obtained by multiplying the maximum number of active blocks by the total memory needed for the samples of a given block, which is (*n × s*) × (*block*_*size × n*). This is precisely the counterpart of this algorithm; despite it is a fast and robust way of updating the background model, it needs to use huge amounts of memory in relation to the size of the images. As an example, the real application updates the background with *n* = 16, *s* = 10 and the block size is *block*_*size* = 2, 560 *bytes* (2 lines of a VGA image in YUV color space). The total memory consumption in bytes is (16 × 10) × (2, 560 × 16) ≈ 6 *Mbytes*. As the Blackfin BF561 processor used has 64 MB of memory, the use of 6 MB is affordable.

### 4.3. Image Labeling

The image labeling phase recovers the binary image created by the segmentation process and produces the first high level data of the scene. Foreground objects are found, labeled and, finally, some features are obtained. Also, Gaussian noise as well as objects not reaching a given size threshold are filtered. The algorithm we have used is a modified version of [23]. It is based on contour tracing technique which allows us to obtain the perimeter, bounding boxes, area and centroid of the objects while labeling them. From now on, we will refer to the elements found during the labeling phase as objects.

### 4.4. Local Object Tracking

The labeling phase assigns ID labels to the objects. The ID of the objects are numbers and are assigned in their order of detection. In addition, objects can have different arrangements from one frame to another. So, the same object can be assigned different labels on different frames. We need to do some additional processing to correlate the objects from the previous scene with the objects from the current scene.

The tracking algorithm is based on the superposition method. Bounding boxes of the objects from the current scene are compared with the boxes from the previous scene. If the boxes are overlapped, a relationship between the objects is considered. This is a low level method and it is not intended for performing complex tracking of people. Currently, high level tracking algorithms use the multiple target tracking methods based on radar. Instead, the tracking for our system has been implemented to solve segmentation errors and enrich the results of the object classification phase.

Tracking the objects on the scene is a complex task. There are several situations that can occur when comparing bounding boxes, from a single match to a multiple match. The main situations considered are the following:

1. An object from the previous scene matches an object from the current scene. Most of the time, the system is going to detect single matches, which are the easiest situation to solve. There is a bounding box on the previous scene that matches a bounding box on the current scene. We just propagate all the information of the previous object to the current object.
2. An object from the previous scene matches several objects from the current scene. This situation can be produced by real segregation of objects (for example two people splitting from a group), or by bad segmentation. To manage this second case, we have tried to manage bad segmentations by introducing an inertia parameter. Inertia is a counter and its objective is to merge objects that split up during a number of frames. During this period the objects will be merged and treated as a single object. If they keep split up for the whole inertia period, then a real split is considered and the objects are separated definitely. If the objects regroup, then the inertia is reset, and this will be considered as a segmentation error.
3. Several objects from the previous scene match a single object from the current scene. The situation can occur either when several objects merge into a group or when bad segmentation was not solved. In either case, the system will never consider the bad segmentation case and will create a grouped object. A grouped object is a new object with new geometrical features, with the difference that it keeps the list of single objects that belongs to it. This way, the system can try to recover the features and classification data from single objects if the group splits up again.
4. Several objects from the previous scene match several objects from the current scene. This is the most complex situation to solve, and we refer to it as a multiple match. In this case, the system simply merges all the objects involved in the multiple match.

As a final consideration, we must assume that the segmentation is not going to perform perfectly in all situations. The system has to be able to reduce the impact of wrong segmentations. Consequently, we must keep in mind that merging objects is always preferable over keeping them split up.

### 4.5. Object Classification

The system requires to classify objects between three different classes: *Group*, *Person* and *Luggage*. Objects are studied taking the information contained into their bounding box. A study based on almost 3,000 objects database has been done in order to obtain the statistical distributions of the features of the objects. From this study, we stated that the features that best distinguish the three classes were the *Number of heads*, the *Filling factor* and the dispersion of the objects, known as *Dispersedness* [24].

To compute the *Number of heads* of an object, the first step is to obtain its upper silhouette. This silhouette is then filtered using a low-pass filter. We have decided to use a mean filter, with a window size proportional to the height of the object, to clear the noise of the signal. Then, the maximums of the resulting signal are obtained, each maximum being a possible head. There is some noise that the filter may not clear, like raised arms, which can be confused as heads. To clear the false heads, the vertical projection of the entire object is used, counting the number of pixels on the column of the maximum. With this feature, we can state a weight for each head and ignore those under an established threshold. In our case, we have established a 40% head weight, which means that at least the 40% of the column containing the maximum on the silhouette has to be filled with object pixels. This so-computed *Number of Heads* allows the system to distinguish *Group* of people from single *Person* and *Luggage*.

The *Extent* or *Filling factor* is the relationship between the area of an object, defined as the number of foreground pixels that contains, and the area of its bounding box:

(4) $$\mathrm{Extent}=\frac{\sum (\mathrm{foreground}\;\mathrm{pixels})}{{\mathrm{BBox}}_{\mathrm{width}}\times {\mathrm{BBox}}_{\mathrm{height}}}$$

This feature gives a measure about how filled is the area of the bounding box of the object. Luggage usually has bigger *Extent* value than people because of its physical properties. For example, the separation between the legs of a person or the fact that the head of the people does not occupy the entire width of the bounding box diminishes the *Extent* for people.

Finally, *Dispersedness* relates the square of the perimeter with the area of the object. A circle is the element with the lowest *Dispersedness*. As the perimeter grows, having the same area, the object becomes more dispersed. As this feature has not an upper bound we have normalized it by using its inverse *(Inverse dispersedness)*. Although the upper bound is undefined, the lower bound is obtained as the *Dispersedness* of the circle, which is the object with the minimum *Dispersedness* possible, being this:

(5) $$\mathrm{Dispersedness}\;\mathrm{of}\;\mathrm{the}\;\mathrm{circle}=\frac{{\mathrm{perimeter}}^{2}}{\mathrm{area}}=\frac{{\left(2\pi r\right)}^{2}}{\pi r^{2}}=4\pi$$

so, to normalize the *Dispersedness* we obtain:

(6) $$\mathrm{Inverse}\;\mathrm{Dispersedness}=\frac{4\pi }{\mathrm{Dispersedness}}$$

Contours of people present usually higher dispersions than contours of luggage, as luggage usually has more compact perimeters.

**Figure 8.**
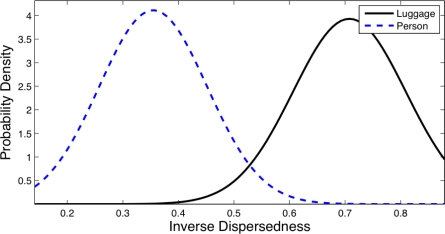
Statistical distributions for *Inverse Dispersedness* feature in *Luggage* and *Person*.

**Figure 9.**
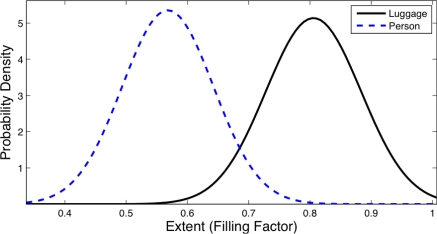
Statistical distributions for *Extent* feature in *Luggage* and *Person*.

Figures 8 and 9 show the distribution obtained from the object database for the *Inverse Dispersedness* and the *Extent* features respectively. For the *Inverse Dispersedness*, the mean values were 0.354 for Person and 0.708 for Luggage, with standard deviations of 0.097 and 0.101 respectively. For the *Extent* feature, the mean values of the measured distributions were 0.567 for Person and 0.805 for Luggage, with standard deviations of 0.074 and 0.078 respectively.

The class of an object is decided probabilistically. The classifier returns a membership probability for each class. This probabilities are merged with the propagated classifier data of the object or, if the object is a new one, assigned directly. To merge the new calculated probabilities with the historical ones of an object, an exponential filter is used. Finally, objects have a membership probability for each class. For the purposes of our system, the class of an object is the class with the highest membership probability.

To assign the membership of an object, a decision tree classifier based on the statistical distributions of the features has been used. This classifier runs very fast, and has very good classification performance, even having ambiguous features. The reinforcement provided by the tracking algorithm allows that isolated bad classifications on single frames do not affect the overall classification.

**Figure 10.**
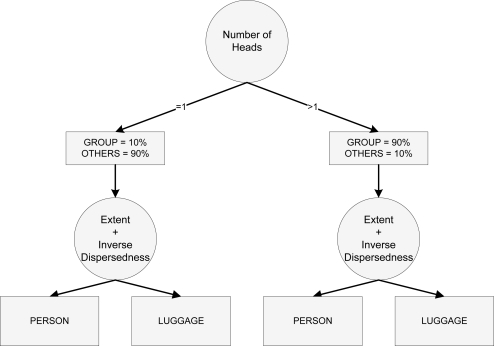
Decision tree used in low-level classification for membership probability assignment. First, the *Number of heads* is used to discriminate the Group. Then, the *Extent* and *Inverse Dispersedness* is used to discriminate between Luggage and Person.

The classification tree is depicted in Figure 10. The number of heads has proved to be very efficient to discriminate in a first step *Group* of people from single *Person* or *Luggage*. If the object has more than one head, then it is considered to be a *Group*. If we find only one head, then the statistical distributions are used to distinguish between *Person* and *Luggage*. Finally, for one headed objects, the mean of the membership results given by the *extent* and *Inverse Dispersedness* features is obtained.

## 5. Experimental Results Obtained

The Quality of Service of the embedded vision system depends not only on the successful detection and classification of the objects of interest, but also on providing enough frame rate to ensure a consistent tracking. In our case, the minimum frame rate required by the upper levels of reasoning was 4 fps. Consequently, our video system was designed taking a higher frame rate as reference: 7.5 fps (due to the timing constraints of the video sensor used). Therefore, we have measured the system performance in terms of algorithm execution times and system classification rates, proving that our system runs at the indicated constant frame rate, and is capable of detecting and classifying objects of interest with a high success rate.

For the experiments, dual core Blackfin BF561 was configured to run at a 600 MHz clock frequency. Algorithms have been distributed over the cores to achieve maximum performance. We have used an Omivision OV7660 camera providing VGA resolution (640 × 480 pixels). On the other hand, compression and Ethernet communication are performed in parallel and its performance is not really interesting so there are no measures presented for the Blackfin BF537.

### 5.1. System Performance

To evaluate the system performance, we have measured latencies for each of the algorithms being executed on the platform. As the platform communicates via Ethernet device, and other nodes are on the same network, frame rate has been limited to 7.5 fps (this means 133 ms per frame). Thanks to the architecture and the vision system design, the node is capable of offering constant frame rate.

**Figure 11.**
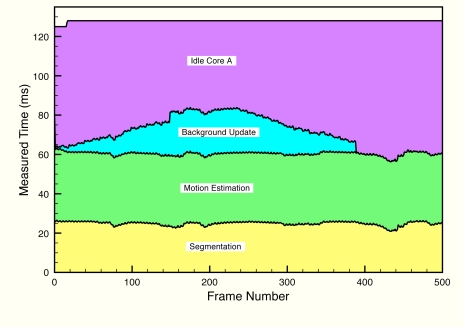
Measured times of each phase of video processing running in Core A, including the idle time. The data are represented stacked. The different areas are labeled with the corresponding phase of processing.

**Figure 12.**
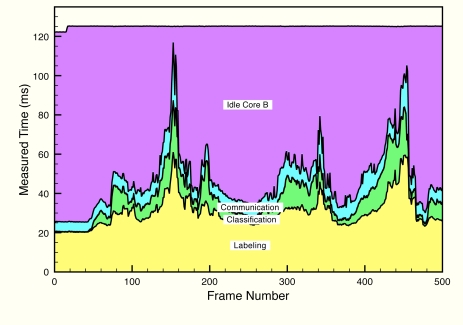
Measured times of each phase of video processing running in Core B, including the idle time. The data are represented stacked. The different areas are labeled with the corresponding phase of processing.

Figures 11 and 12 depict execution times taken during a test on a real scenario. In the figures, execution times are presented stacking the processing times of each algorithm for each frame. In addition, idle times have also been measured (they have not been calculated offline), proving that the system runs at constant frame rate.

As seen in Figure 11, foreground and motion detection times are almost constant. The most interesting measure of core A is the one corresponding to the background update process. This background processing time corresponds with the processing scheme shown in Figure 6. This measure proves that our background update algorithm is capable of running in real time without a severe impact on processing times. Also, as the updated background model does not depend on previous background model values, it has proven to be a very robust algorithm on very long term executions.

For the processing times on core B, we must take into account that predicting execution times accurately on vision systems is extremely difficult, as the list of possible different situations in relation to the geometry and size of the objects on scene is almost endless. However, there is a relationship between processing times and the total area the objects occupy on scene. Core A is not affected by this fact because, precisely, it is detecting foreground pixels, and the mathematical operations are the same for each pixel, be it a foreground or a background pixel. On core B the situation is different. If the total area is bigger, labeling has to find more elements and classification involves evaluating larger areas on the scene. In [23], a proof is given that, while labeling the frame, each pixel is not visited more than four times. Also, classification has been implemented in a way that each pixel is visited only once. Therefore, the number of times a pixel is visited is bounded and execution time is bounded as well.

**Figure 13.**
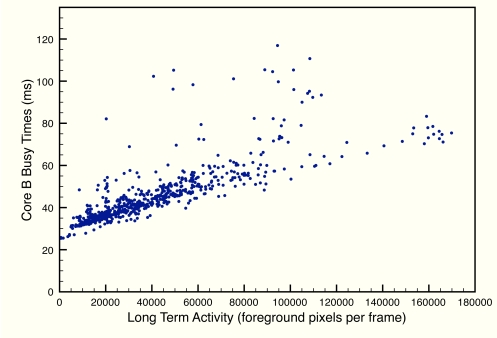
Measured processing times of the Core B as a function of the Long Term Activity (number of foreground pixels) of each frame, during a real sequence with 750 consecutive frames.

We have evaluated the worst case execution time to find out if the system can really run at a constant frame rate regardless of the number of foreground pixels. Figure 13 depicts the relationship between the Long Term Activity (number of foreground pixels) and execution times. As seen in the figure, even on frames with large foreground pixel occupancy, the times do not reach the 133 ms given by the 7.5 fps limit.

Finally, the latency between the real event and the detection of the situation is also constant. The system has three parallel processing units and, as stated, the response time for each unit is 133 ms. Thanks to the designed pipeline, there are no queues, so each scene will remain on the system the sum of the latencies of the processing units, which is 399 ms. In conclusion, objects of interest will always be detected and registered in less than half a second.

### 5.2. Classification Rates

To evaluate the real behavior of the prototype, a short real sequence was taken over 3,000 frames. The obtained classification results as well as the images have been recorded and the classification results have been inspected frame-to-frame in a manual way. From this short scene, a total of 643 persons, 202 groups and 390 luggages were detected and classified by the prototype. The classifications results obtained have been summarized in the Table 1.

Most of the experiments done are aimed to test the quality of the final classification of objects, as this is the goal of the complete process. The current results are satisfactory, but improvements are still possible and desirable. Thus we continue working on these issues, enhancing the current silhouettes databases and obtaining more videos with ground truth, to make better evaluation tests.

## 6. Conclusions

We have presented the details of the implementation of the SENSE vision system. This paper shows the large number of implementation challenges with which a vision system must deal. Not only the vision algorithms must meet the minimum requirements, but also the performance of the whole system must be good enough to infer a consistent environment model. Thus, the success of the system depends on linking the set of vision algorithms with a good set of architecture management algorithms.

Classification results, as being the output of the process, may be measured as a whole, considering the total amount of objects expected to be segmented, tracked and classified as a single stage. The results obtained in our real experiments seemed very satisfactory, as expected from our previous off-line experiments with Matlab environment. Table 1 summarizes the classification results obtained for a single experiment under real conditions. However, for all off-line tested videos, the True Positive rate in all classes has been higher than 80%, being this the limit agreed to ensure high level robustness.

## Figures and Tables

**Table 1.** Classification results obtained with the implemented software during a real experiment.

**Object Class**	**Classified as:**		
	**Person**	**Group**	**Luggage**
Person	91.9%	19.3%	0.3%
Group	6.8%	80.7%	0.5%
Luggage	1.3%	0.0%	99.2%
